# Face Value: Beauty, Punishment, and the Moral Politics of Appearance

**DOI:** 10.3390/bs15121717

**Published:** 2025-12-11

**Authors:** Franziska Hartung, Maxime Levasseur, Ewan J. Lomax, Gareth Richards

**Affiliations:** School of Psychology, Newcastle University, Newcastle upon Tyne NE2 4DR, UKewanlomax1@gmail.com (E.J.L.); gareth.richards@ncl.ac.uk (G.R.)

**Keywords:** facial disfigurement, punitive mutilation, facial attractiveness, social stigma, acid attacks, punishment, facial difference, visible difference, disfiguration

## Abstract

Faces are central to human interaction, serving as primary sources of identity, emotional cues, and social judgments. Facial attractiveness is strongly linked to perceptions of trustworthiness and moral goodness, leading to preferential treatment across education, employment, and legal contexts. Deviations from facial norms—such as asymmetry or visible differences—are, by contrast, often associated with negative traits, social avoidance, and dehumanisation. Across cultures and centuries, deliberate facial disfiguration has been used as a form of punishment for perceived moral or legal transgressions. Evidence from ancient Egypt, Mediaeval Europe, and early modern legislation, as well as modern acid attacks, indicates that intentional facial disfiguration has long served as a means of ongoing punishment through humiliation and identity disruption. Motivations for targeting the face may be rooted in its central role in identity, beauty, symmetry, and symbolic purity. Despite contemporary legal efforts to curb acid attacks and related violence, legislation specifically addressing intentional facial disfiguration remains limited. Modern psychological research confirms that acquiring a facial difference can severely impact quality of life, social functioning, and identity. This paper synthesises historical, cultural, and psychological perspectives on punitive facial disfiguration, highlighting its enduring role as a mechanism of social control. Future research should examine perpetrators’ decision-making, possible differences between different types of facial disfiguration, and the perceptual and emotional consequences of different facial injuries to inform prevention strategies and improve support for victims.

## 1. Introduction

Faces play a fundamental role in human interaction and perception, often serving as our primary source of information about others. They are crucial for recognising identity and emotions (De Sonneville et al., 2002) and for inferring people’s intentions and interests (Crivelli & Fridlund, 2018). Faces are more salient than most other stimuli (Keys et al., 2021). Our perceptual system is so tuned to detecting faces that we frequently perceive them in non-face objects—a phenomenon known as face pareidolia. This tendency facilitates objects with illusory faces being detected more rapidly than those without (Keys et al., 2021) and is thought to be a byproduct of the evolutionary pressure for rapid face detection, which aids in identifying others and avoiding potential threats (Caruana & Seymour, 2022).

The development of social cognition critically relies on processing faces, with this skill beginning in early infancy. Faces are among the most common and salient stimuli in a newborn’s limited visual world (Fausey et al., 2016). Infants are naturally drawn to faces over other objects (Morton & Johnson, 1991; Gamé et al., 2003; Macchi Cassia et al., 2004; Kwon et al., 2016) and remember them better (Bahrick et al., 2002). This preference is driven by the typical arrangement of facial features, especially the eyes and mouth (Morton & Johnson, 1991; Farroni et al., 2005). Exposure to facial features and facial expressions within sensitive developmental periods is crucial for the development of the neural networks that support social cognition (Johnson, 2005).

From an evolutionary perspective, faces also play a role in choosing partners, as facial attractiveness has been proposed to signal health (Rhodes et al., 2007; Henderson & Anglin, 2003). Recent research has questioned the link between health and attractiveness as being weak and unreliable (Foo et al., 2017; see also Talamas et al., 2016). However, there is surprisingly little cross-cultural variation in attractiveness judgments, particularly at the extremes of low and high attractiveness (Little et al., 2011; Pavlovič et al., 2023), suggesting that biological factors play a significant role. Well-established predictors of facial attractiveness include symmetry, proximity to the population average, and sexual dimorphism (Rhodes, 2006; Rhodes et al., 2007). However, these factors account for less variation in attractiveness ratings than previously thought (Jones & Jaeger, 2019), indicating that facial attractiveness judgments are more nuanced and complex than traditional evolutionary theories have proposed.

Attractiveness judgments are made rapidly (Kaiser & Nyga, 2020) and are strongly correlated with social evaluations, particularly perceptions of trustworthiness (O’Doherty et al., 2003). More importantly, these judgments appear to be robust to change, as prolonged exposure to a picture of a face seems to consolidate people’s confidence in initial impressions (Todorov et al., 2009; see also Hu & O’Toole, 2023). High morality, trustworthiness, goodness, and talent are often attributed to people with beautiful faces, observations which are sometimes explained in terms of the halo effect (Dion et al., 1972). In contrast, people with unattractive or anomalous faces are often assessed as having poor character or personality traits and a lack of competence and trustworthiness (Jamrozik et al., 2017). This association between beauty and morality has direct consequences for people’s lives. Research shows that attractive people are often treated more favourably in nearly every area of life—from school and work to friendships and even family—and given lighter prison sentences for the same crimes (Dion et al., 1972; Todorov et al., 2009; Stewart et al., 2012; Langlois et al., 2000; Zebrowitz et al., 2002), reflecting a widespread association that beauty signals goodness and potential.

While there is much research on the benefits of facial beauty (e.g., health and social advantages), much less has been conducted regarding the effects of facial abnormalities. Similarly to the beauty is good (and unattractive is bad) stereotype (Dion et al., 1972; He et al., 2022), there seems to be an extension of this stereotype, or an additional stereotype, indicating that divergence from facial norms is detrimental. Faces with visible differences are typically asymmetric and deviate from facial averageness, meaning they lack the average facial features within a given population. While attractive faces elicit neural reward responses (Pegors et al., 2015; Aharon et al., 2001; Cloutier et al., 2008), faces with visible differences are linked to saliency responses in perceptual areas and reduced activity in social brain areas (Hartung et al., 2019; Workman et al., 2021), suggesting social disengagement and possible dehumanisation. Consistent with this, eye-tracking research has shown that faces with visible differences draw prolonged attention due to their distinctiveness and saliency (Halioua et al., 2011) but disrupt typical gaze patterns, diverting attention from socially relevant cues like the eyes (Rasset et al., 2022; Ishii et al., 2009; Meyer-Marcotty et al., 2010; Villavisanis et al., 2023). This disruption can impair emotional and social processing, reinforcing negative emotional responses (Rasset et al., 2022, 2023; Stone & Potton, 2014). Individuals with strong implicit bias often avoid looking at anomalous faces (Villavisanis et al., 2023), though avoidance is absent during covert attention (Boutsen et al., 2018, 2022), meaning that, while staring is inhibited, attention is still high. Implicit bias is particularly high among those with strong just world beliefs—the idea that people get what they deserve (Lucas et al., 2011; Workman et al., 2021).

Facial differences elicit swift, biassed inferences that often lead to real-world discrimination in social, academic, and professional contexts (Saunders, 2019, 2020). Individuals with facial differences are frequently rated as less attractive and less desirable as romantic partners (Jamrozik et al., 2017). Even minor scars can lead to perceptions of reduced sociability, attractiveness, and honesty (Bull & David, 1986). Viewing a face with visible differences tends to reduce observers’ feelings of happiness, control, and dominance, while increasing physiological arousal (Jamrozik et al., 2017). Beyond these immediate reactions, facial differences are frequently linked to perceived unfavourable personality traits (e.g., low emotional stability, low conscientiousness), negative internal attributes (e.g., unhappiness, reduced intelligence), and adverse social qualities (e.g., untrustworthiness, unpopularity; Jamrozik et al., 2017), all of which reinforce discriminatory treatment in everyday interactions. As a result, having a facial difference is linked to overall lower quality of life (Somoye et al., 2021).

Some of the associations between attractiveness and adherence to norms with perceptions of (moral) goodness may be based on an inherent bias in face processing stemming from evolved mechanisms, such as pathogen avoidance (Oaten et al., 2009; Schaller & Duncan, 2007), kinship normativity (J. H. Park & Schaller, 2005; Hansen et al., 2020), and preference for potential markers of biological and reproductive health (Little et al., 2011). However, the impact of culture and media on reinforcing stereotypes should not be underestimated. In Western culture, facial differences are often used to visually distinguish good and evil characters in media productions (e.g., Bond villains), especially media aimed at children, such as Disney productions (Croley et al., 2017). In many visual narratives, the transition of the moral alignment of a character is marked by the acquisition of a visible difference (e.g., Anakin Skywalker, TwoFace) or a significant change in attractiveness (e.g., Nanny McPhee). Even outside of Hollywood, this stylistic device is common, with examples including the famous Nollywood actress Iya Gbonkan, who, due to her facial scarring, is frequently cast as a witch, and the former Bollywood actor Jeevan, who, with a prominent mole on his face, was regularly cast as a villain. Interestingly, there is some recent evidence that little to no exposure to media perpetuating these stereotypes is associated with less bias against faces with visible differences and that bias increases with media exposure (Workman et al., 2022). However, this evidence is tentative and has only been shown for facial scarring, which, as an acquired injury, may be based on different associations than congenital (e.g., cleft palate) or health-linked facial differences (e.g., skin cancers or facial palsy).

While negative stereotypes of people with visible differences are prominent in modern media productions, it is incorrect to assume that this is a modern development. In Grimm’s fairy tales, witches and trolls, evil stepmothers, and other villains have frequently been depicted as unattractive and/or as having visible differences, and many ancient myths communicate the stereotype that the beautiful are good and flaws in appearance reflect questionable morals (Baker-Sperry & Grauerholz, 2003). This is also true outside of Western cultures, for example, in African art, mythology, and beliefs about witches (Adinkrah, 2015; Cowcher, 2012; Petridis, 2022; Roxburgh, 2016; Van Damme, 1991). In fact, many cultures—present and past—not only associate ‘unattractive’ people, or people with facial differences, with moral flaws, but also have (illegal) practices or even laws that involve punishments to permanently alter the facial appearance of people who are considered moral violators in their society. Historically, we can see evidence of the legal practice of punitive facial disfiguration (Skinner, 2015), as well as the facial branding and head-shaving of women who were accused of affiliating with the enemy during wartime (Virgili, 1995; Škodrić, 2015; Ericsson, 2016). However, even in present times, facial mutilation is surprisingly common, within and outside of legality: for example, vitriol (sulphuric acid) crimes as a form of vengeance and humiliation to (often) literally deface women accused of infidelity, non-reciprocal lovers, and assumed members of opposing gangs (e.g., Acid Survivors Foundation (Bangladesh), 2018; Yeong et al., 1997; Byard, 2020). The following explores punitive facial disfiguration resulting in acquired facial differences as an expression of normative cultural aesthetics and relates this practice to the previous literature linked to the importance of faces. Across historic and cultural contexts, we evidence practices and apparent motivations behind the intentional infliction of facial disfigurement and discuss potential preventative legislation.

## 2. Historical Evidence of Facial Disfiguration as Punishment

The earliest evidence of intentional facial disfigurement comes from pre-pottery Neolithic crania, found at Tell Qarassa North (8500–8300 BCE). Santana et al. (2012) report several distinct types of postmortem facial mutilations, including the removal of, or severe damage to, key facial structures including the nasal region, upper jaws, and cheekbones, as well as signs of defacing of the eye sockets and mouth area, alongside cuts and tool marks. Unlike other Neolithic sites, where skulls were plastered and reconstructed, presumably to honour the deceased, these skulls were stripped and defaced rather than beautified. While we have limited knowledge about punishment practices in the Tell Qarassa culture, Santana et al. (2012) interpret the mutilation as possibly reflecting punitive or symbolic hostility.

The punitive disfigurement of nose and ears has been evidenced as a judicial punishment across many cultures, with the nose being the most common target (Sperati, 2009). In the pre-Mediaeval period, removal of the ears and nose was common as a punishment in the New Kingdom Egypt (1550–1070 BCE). Egyptian nose and ear removal was a complex practice, characterised by multiple layers of enforcement and meaning. It was often threatened but not always carried out. It has been interpreted as a punishment, as the mutilation of sensory organs was thought to prevent the individual’s spirit from breathing and functioning in the afterlife. As the body was believed to transition into the afterlife in its most recent state, it was thought that this would hinder interaction with the divine, disrupting the receiving of wisdom or judicial mercy (Loktionov, 2017). Within this context, the removal of noses and ears is predominantly interpreted as a form of disablement rather than disfiguration. Nasal removal has also been observed in Mediaeval Spain, Novgorod (the Russian state from the 12th to 15th century), and the Holy Roman Empire (Byzantine) suggesting that this practice was seen as a common and accepted form of punishment during the Mediaeval period (Skinner, 2015).

Blinding and nose removal were common for political opponents in the Byzantine empire, alongside castration and limb amputation (Longworth, 1997; Turner, 2024). Here, the rationale was directly and openly linked to the threat perceived from those individuals. Within Byzantine political and theological ideology, the emperor was expected to reflect divine perfection; consequently, physical integrity was considered essential for legitimate rule. Bodily mutilation, particularly of the face, was regarded as incompatible with imperial authority and thus rendered an individual ineligible to ascend the throne (Ostrogorski, 1957; Stumpf, 2017). An exceptional case is that of Justinian II (“the slit-nosed”), who, despite suffering nasal amputation following his deposition in 695, successfully reasserted his claim as emperor in 705 (Ostrogorski, 1957).

Archaeological evidence from a young woman who lived in Anglo-Saxon England during the Mediaeval period shows removal of the nose, lips, and forehead (possibly from scalping), indicating punitive intent (Cole et al., 2020). Although written records of facial mutilation as punishment do not appear until the tenth century AD, facial disfiguration practices as punishment may have emerged at least a century earlier. Facial disfiguration was used to punish a range of crimes, though the specific methods and facial features that were targeted varied. King Edmund’s (920–946) third law code specified slaves should be punished through removal of the scalp for theft. This law was extended by King Edgar (959–975), with tongue removal serving as a punishment for making false accusations (Cole et al., 2020).

Similarly to early Mediaeval Europe, early Mediaeval India (1st–11th centuries CE) had legal codes including penalties like blinding, cutting off the nose or ears, branding, and other disfiguration punishments as alternatives to capital sentences for crimes such as theft, adultery, assault, or treason (Skinner, 2015). Mutilation was often framed as a lenient alternative to death, though it was understood to carry permanent stigma and social marking (Skinner, 2015). Early Mediaeval Indian law codes (e.g., Dharmashastra, Manusmriti) included more frequent and detailed prescriptions for disfiguration, particularly nose-cutting, as punishment for adultery or sexual transgressions. While punitive disfiguration was described in both early Mediaeval European and Early Mediaeval Indian law codes (e.g., Anglo-Saxon, Carolingian, Byzantine law), it was less commonly enacted in Europe than in India; instead, threats or fines often replaced physical penalties (Skinner, 2015).

The reasons for facial disfiguration against women in Mediaeval times were heavily linked to sexuality (Skinner, 2015). Women were punished with facial disfiguration for (alleged) adultery, evidenced, for example, by King Knut’s (1016–1035) 2nd law code detailing the punishment for a woman accused of adultery as removal of the nose and ears. Even in more recent history, we see similar motivations, with the head-shaving and facial branding of women who had sexual relations with German soldiers in post-WW2 France (Virgili, 1995), Serbia (Škodrić, 2015), and Norway (Ericsson, 2016). These acts may symbolise male power and social control rather than represent an expression of legal or communal sanction. However, while the punishment itself may be intended to inflict shame, there was some debate about the perception of women with facial differences during the later Middle Ages, with responses ranging from sympathy to repulsion, judgement, and rejection (Skinner, 2015).

In the United States, Jefferson’s provision for facial disfiguration in Bill 64 (Jefferson, 1950) described facial maiming as a moral transgression and proposed retaliation (“an eye for an eye”) as appropriate punishment (“proportionate maiming”). More specifically, retribution was considered to comprise equal disfiguration and the forfeit of land and goods (Cock, 2019): “Whosoever shall maim another, or shall disfigure him by cutting out or disabling the tongue, putting out an eye, slitting the nose, cutting or biting off a nose, ear, or lip, branding, or otherwise, shall be maimed or disfigured in like sort; or if that cannot be, shall forfeit treble damages to the party injured.” (Jefferson, 1950). Jefferson’s provision in Bill 64 also proscribed removal of the nose as a punishment for women guilty of rape, polygamy, or sodomy, due to its high visibility, which made offenders easily identifiable and brought shame, while men were to be castrated for these crimes (Cock, 2019). Punitive disfigurement was framed in this bill as a more lenient punishment as compared to the death penalty.

The types of intentional facial disfiguration inflicted started to evolve in the early modern period, with acid attacks becoming more common in the UK due to sulphuric acid being manufactured at an industrial scale since the 1740s (Anderson, 2013). In East Asia today, facial injuries resulting in facial differences are often the result of chemical assault (vitriolage). Data on acquired facial differences following vitriolage show that sulphuric acid was used in 90% of reported cases (Yeong et al., 1997).

## 3. The Motivations Behind Facial Mutilation as a Punishment

The motivation for inflicting permanent facial differences is often—but not universally—linked to sex/gender. In South Asia, the Acid Survivors Foundation (https://acidsurvivors.org/, accessed on 24 October 2025) highlights the major reasons for acid attacks as the refusal or rejection of love (20%) and family-related disputes (20%). Almost 80% of victims are women, and 40% of perpetrators are the victim’s spouse (Yeong et al., 1997). In Bangladesh, vitriolage usually seems to be motivated by the refusal of sexual advances, and marital affairs, with this violence mostly being toward young women. Similar findings have been reported by Kamruzzaman and Hakim (2016), who reviewed acid crime in South Asia and highlighted causes including dowry, family dispute, property dispute, marital dispute, and refusal of love. Converging evidence has been reported by the Acid Survivor’s Foundation and an integrative review by Kornhaber et al. (2023) of violence by burning (including chemical burns from vitriolage) against women across nine countries. Data reviewed from India, Bangladesh, Nepal, Iran, Jordan, Pakistan, Brazil, Colombia, and Cambodia emphasise gendered motivations for violence, such as the denial of sexual advances or marriage proposals, marital conflict (including jealousy, infidelity), and dowry-related violence (especially in India and Bangladesh), with (young) women being targeted most frequently (Kornhaber et al., 2018). Another common motivation are land and property disputes (Kornhaber et al., 2018). Revenge and humiliation are highlighted as key motivations. When women were the targets, the most common anatomical areas targeted were the head, face, and neck. Similarly, data from Colombia show a trend of perpetrators predominantly being men and victims predominantly being women (Romero-Chacón et al., 2025).

Some data from other geographical areas do not show that women are more frequently victims. For example, data from Uganda reveal little difference in the number of acid attack victims in respect to gender (Asaria et al., 2004; Acid Survivors Foundation (Bangladesh), 2018), whereas in Nigeria more victims are men (Mannan et al., 2007). In Jamaica, which has some of the highest rates of acid crimes, women seem to be common as both victims and perpetrators (Branday et al., 1996). In contrast, the victims and perpetrators of acid attacks in the UK are predominantly male, with vitriolage being mainly associated with gang violence as a means to punish or intimidate (Byard, 2020).

While there are significant cultural differences in the choice of methods for inflicting facial injuries, and in the relationships between victims and perpetrators, facial disfiguration with the goal to punish seems to be present in many cultures. In contrast to historical instances of facial disfiguration, which often occurred as a punishment for moral transgression and were regulated by law, modern facial disfiguration may more frequently stem from a motivation for revenge for perceived moral transgressions on a personal level. Many cases appear to occur in the context of romantic or sexual disputes, or are carried out with the intention of humiliating members of opposing gangs. In general, the aim of facial disfiguration appears to be ongoing punishment through humiliation, rather than hiding an intention to kill (Brady, 2013; Farhad et al., 2011; Frembgen, 2006; Skinner, 2014).

The consequences of acquiring facial differences through intentional violence are described by victims in terms of alienation, recurring (physical, emotional, and social) loss, existential efforts to sustain identity, and novel perspectives on life (Martindale & Fisher, 2019; Konradsen et al., 2012; Costa et al., 2014). Although, previously, the negative effects on victims were seen as intuitively true, there was no empirical evidence characterising the lived experiences of victims until relatively recently. Victims of facial trauma have a high risk of anxiety and depression (Islam et al., 2012). Qualitative research on women who were victims of facial disfiguration has found recurring themes, including physical appearance being related to codes of stigmatisation and negative perception by others (Habib et al., 2021). A study conducted in India on the experiences of victims of intentional facial disfiguration through immolation found everyday stigma, threats to family life, and threats to religious sensibilities (Furr, 2014). Moreover, data from South Asia showed varying levels of disgust in response to facial differences, ranging from pity to fear (Habib et al., 2021).

The previous literature has highlighted the historical and cultural perspectives of facial disfiguration, but there is an important psychosocial impact that should also be addressed. A prominent historical example from the UK is the “blue benches” near Queen Mary’s Hospital in Sidcup, which subtly identified World War I veterans with facial differences and provided them with a space where the public would not stare—recognising the difficulty of their return to everyday life (Biernoff, 2011). Fear of rejection is a psychological outcome experienced by many victims of acid attacks (Mittal et al., 2021). Receiving correctional treatment for facial differences seems to increase extraversion, openness, agreeableness, conscientiousness, and self-concept, as well as reducing self-consciousness (Mendes et al., 2019). Having a facial difference is linked to lower quality of life (Somoye et al., 2021). More specifically, having an acquired facial difference has been linked to conflicting interpersonal relationships and poorer work ability, as well as poorer quality of life and poor access to treatment (Reis et al., 2018). People with acquired facial differences tend to have a lower educational level and income, and lower quality of life and Work Ability Index scores, than people with congenital deformities affecting the face (Reis et al., 2018; Saunders, 2019, 2020). However, after treatment to remove a facial difference, no significant differences are found in the overall quality of life or quality of health between people with and without facial differences (Somoye et al., 2021), suggesting that it is the presence of the facial difference alone which significantly impacts the life of an individual. The causal relation between quality of life and facial differences may underline the motivation for using facial disfiguration as an ongoing punishment. This is because there is a clear negative psychosocial impact to having a facial difference, manifesting in enduring social alienation and stigmatisation from others (Habib et al., 2021), which the victim would suffer from upon acquiring a permanent facial difference.

What are the psychological processes behind punishment via facial disfiguration? Through the lens of historical law, facial disfiguration was mostly interpreted as allowing for the easy identification of offenders, and as bringing shame for their transgressions (Cock, 2019). A recent interview study highlighted that faces are one of the main influences in identity creation, and that disfiguration may therefore be a way to disrupt this sense of identity (Martindale & Fisher, 2019). This links to evidence from Egyptian statues, where defacement acted as a ritual deactivation by symbolically taking away the statues’ senses (Connor, 2019). While this behaviour could also have reflected a shift in religious beliefs at the time, the act of defacing statues may be considered as further evidence of harming identity through facial disfiguration. Similarly, Romans extended punishment beyond death by defacing both corpses and representational “bodies” (portraits and statues). This functioned as a public, performative ritual that humiliated, delegitimised, and erased leaders fallen into disgrace or political enemies from the civic community (Varner, 2001, 2020) while warning onlookers about the consequences of treason and tyranny.

It has been found repeatedly that the nose is a target of facial disfiguration, possibly because it is an important and salient part of the face, located right in the centre (Cole et al., 2020). Research specifically investigating punitive nasal removal has highlighted the sexual symbolism of the nose in several cultures, including in India, Germany, and the Punjab, as well as in various folklore (Frembgen, 2006). Cultural differences in beauty standards may also play an important role. For example, a long slender nose is considered beautiful in Pakistan, particularly for women. In Pakistani culture, nasal disfiguration is sometimes performed as retaliation, to balance a debt of honour—by cutting off the adulteress’ nose, a man may be considered to imprint his power onto a woman’s body (Frembgen, 2006). These motivations are also reflected in pre-Mediaeval law code, where adulteresses’ noses were removed as a form of punishment (Skinner, 2015). The consistency of nasal removal to punish moral transgressions affecting purity specifically is interesting and prompts questions for future research into the impact of the placement of the facial difference on a viewer’s perception and judgement. Frembgen (2006) concludes that the practice of nasal removal is driven by society and politics—if a society places greater value on body purity, this is thought to lead to increased rates of nasal disfiguration towards alleged adulteresses; similarly, increased rates of nasal removal towards men, for degradation and trophy-hunting purposes, is predicted in countries that are more war-torn.

Another potential explanation for why facial disfiguration as a form of punishment is present across many cultures and time periods relates to the value of facial symmetry. Facial symmetry is strongly linked to perceptions of attractiveness and trustworthiness (Rhodes, 2006) as a proxy for moral goodness. Countless film productions in which villains are displayed with facial differences, like Scar in the Lion King, reflect this perceived link between beauty and morality. In masks of the Chinese Opera, and the Dan culture of Western Africa, asymmetric masks represent ugliness and moral failing (Scheuerle & Firth, 2021). However, counterexamples also exist. For instance, the Nazca and the Wari in South Peru are cultures in which figures with facial differences depict strong characters with facial injuries (Scheuerle & Firth, 2021).

In many cultures, women comprise the majority of victims in modern day cases of intentional facial disfiguration (Yeong et al., 1997). This might, therefore, suggest another potential motivation behind facial mutilation as a form of punishment. Women have always been prized for their beauty, with facial attractiveness being valued more highly in women than in men (J. Park, 2019). Therefore, the fact that, at least in some cultures, the majority of intentional facial disfiguration cases affect women as victims highlights a form of gendered punishment, whereby a woman’s beauty is mutilated or taken away from her. The threat of physical violence in general may act as a means by which males have reduced the likelihood of infidelity in their female partners. Although speculative, the threat of facial disfiguration could potentially act as a particularly strong deterrent in this regard, due to the social costs being so high. This idea may be consistent with other sexually differentiated patterns of human violence that have been reported, such as mariticide (i.e., the killing of a husband) being less common than uxoricide (i.e., the killing of a wife). Daly and Wilson (1988, p. 202) noted “In every society for which we have been able to find a sample of spousal homicides, the story is basically the same: Most cases arise out of the husband’s jealous, proprietary, violent response to his wife’s (real or imagined) infidelity or desertion.” It should also, of course, be noted that explanations for the occurrence of such behaviours do not represent justifications.

Despite the continuing prevalence of facial disfiguration, many countries have legislation in place to reduce incidence rates. In the UK, for example, the 2018 Tightening of the Poisons Act (1972) attempted to reduce the risk of disfiguration by acid attacks by banning people from possessing sulphuric acid above 15% concentration without a valid reason. In 2019, the UK also established the Offensive Weapons Act (Offensive Weapons Act, 2019), with an entire section outlining rules regarding corrosive products and substances, precluding their sale to under-18s, and banning anyone from carrying such substances in public. This should make facial disfiguration through acid attack more difficult to achieve, thus hopefully reducing incidence rates. Similarly, in Pakistan, the 2011 Acid Control and Crime Prevention Bill was passed, which recommended a 14-year to lifetime imprisonment sentence and high fines for vitriol crimes. Bangladesh also passed the Acid Crime Control Act in 2002, its first legal framework to prevent acid offences. According to some sources, these measures led to sharp decreases in vitriol crimes, suggesting that substance control is critical for harm prevention (Metropolitan Police Service, 2025). However, other sources indicate another rise in vitriol incidents, questioning the effectiveness of substance control alone in preventing vitriol crime (Siddique, 2024; Hopkins et al., 2022). Other methods of inflicting facial differences are less manageable via substance control, and legislation specifically outlining permanent facial disfiguration as a crime is missing. A legal framework informed by empirical evidence is needed to discourage facial disfiguration and deliver justice to victims. More research into this area to understand the cognitive processes behind perpetrators’ decision-making before an attack may help to reduce the prevalence of violent facial disfiguration.

While we reviewed both historical and current data from many cultures across the world, we are limited by the availability of data and existing historical accounts. As a result, we have notable gaps in both geographical regions (e.g., South-East, Central, and North Asia, Oceania, Pacific Islands) and historic periods, limiting the generalisability of our conclusions. From the literature that was available, however, we can deduce apparent trends in motivations for inflicting facial disfigurement with punitive intent. First, while violence is a central motivation, there seems to be the intention that the victim survives the injury. Second, intentional injury is focused on visible areas of the face that are difficult to hide from public view, possibly to inflict ongoing negative social consequences for victims, such as social isolation and humiliation. In addition, we speculate that there may be an underlying motivation of defacing a victim’s identity which links the perceptual processing of faces to mechanisms of dehumanisation.

## 4. Conclusions

Faces are at the centre of human interaction and are often our primary source of information about other people. The perception of facial norms is rooted in cultural values and often linked to perceptions of morality. In this paper, we outlined a brief historical overview of intentional facial disfiguration as a form of punishment. Across time and cultures, beauty and symmetry have been read as signs of virtue, while facial difference has been treated as a shorthand for vice, danger, or diminished worth. Against this backdrop, punitive facial disfiguration may have emerged not as an attempt to kill but as a deliberately enduring sanction. Although there have been developments in the type of method used (such as the use of sulphuric acid in modern times) and certain cultural differences, facial disfiguration seems to have its roots as a form of ongoing punishment for perceived moral transgressions, with the goal of signalling moral flaws and socially isolating transgressors. Currently missing in the literature are accounts providing a more nuanced understanding of different types of visible differences and public beliefs about these conditions and their causes (e.g., acquired vs. congenital, pathological vs. trauma-inflicted). As a result, much of the heterogeneity of facial conditions is difficult to interpret through a cultural lens.

As humans, we use faces to infer social, cultural, moral, and reproductive value via aesthetic judgements. Inflicting permanent damage to faces is an ancient means of punishment for perceived moral transgressions that is used to identify, humiliate, and reduce perceived value. In this way, facial disfiguration intentionally links aesthetics to social regulation through signals to others in a social group. Marking faces as norm-divergent is used to signal moral transgression. Deviation from the norm is recognised as a moral warning signal, while adherence to aesthetic norms signals moral value. This moral signalling is prominent in storytelling practices contrasting beautiful heroes with norm-violating villains, symbolically linking aesthetics and moral transgressions. The often-gendered pattern further indicates that punitive facial disfiguration frequently functions as a tool of social control over women’s sexuality, desirability, and autonomy. Cultural symbols (e.g., the nose as a site of honour, purity, or sexuality) and aesthetic norms (symmetry and averageness) may help explain both target selection and the stigma that ensues.

Although future work should continue to consider victim outcomes, it should also examine perpetrator decision-making and the socio-cultural motivation for targeting faces specifically. Better understanding of the phenomenon of intentional facial disfiguration may help inform judicial and public health policies. Prevention should go beyond supply-side controls (e.g., corrosive regulation and tracking), and could possibly legally recognise facial disfiguration as an aggravating or stand-alone offence that causes lasting psychosocial harm. Clinical pathways could integrate acute care, reconstructive options, and routine screening for mental health and social functioning, alongside evidence-based psychosocial interventions.

Public education and media literacy may help decouple moral worth from appearance and reduce the biases that marginalise people with visible differences. Moreover, careful consideration should be made in terms of media aimed towards children and youth that perpetrates and reinforces harmful stereotypes. Although more research may be required for firm conclusions to be drawn regarding efficacy, this approach might have potential to help reduce the stigma of facial difference. If so, it could make intentional facial disfiguration a less effective punishment, and, perhaps, reduce the overall burden of stigma for people with visible facial differences.

To obtain a better understanding of the impact of stigma relating to facial differences, we should explore relevant data more systematically and with more nuance, alongside other demographic variables such as gender and disability status. While often treated as a homogeneous construct, facial differences vary greatly, and we should explore how injury type, location, and severity shape observers’ perceptions and emotions. To better understand the impact of evolutionary mechanisms, we need to distinguish the social consequences of congenital versus acquired differences and evaluate policy and clinical interventions longitudinally. An interdisciplinary linking of medical and psychosocial outcomes with the biological principles that underlie the social bias approach may increase our understanding, and lead to improved prevention strategies and support structures.

## Data Availability

Not applicable.
